# A Randomized Controlled Trial of Two Different Lengths of Nicotine Replacement Therapy for Smoking Cessation

**DOI:** 10.1155/2013/961751

**Published:** 2013-09-09

**Authors:** Abu S. Abdullah, Anthony J. Hedley, Sophia S. C. Chan, Tai-Hing Lam

**Affiliations:** ^1^School of Public Health, Guangxi Medical University, Nanning, Guangxi, China; ^2^Department of Medicine, Boston Medical Center, Boston University Medical Campus, Boston, MA 02118, USA; ^3^Department of Community Medicine, School of Public Health, Li Ka Shing Faculty of Medicine, The University of Hong Kong, Hong Kong; ^4^School of Nursing, Li Ka Shing Faculty of Medicine, The University of Hong Kong, Hong Kong

## Abstract

This study examined if 2-week free nicotine replacement therapy (NRT) would be more effective than 1-week free NRT to help smokers quit smoking at 6 and 12 months. In a single-blinded randomized controlled trial design, 562 Chinese smokers who attended a smoking cessation clinic in Hong Kong, China, were randomly allocated into two groups (A1 and A2): A1 (*n* = 284) received behavioural counselling with free NRT for 1 week; A2 (*n* = 278) received similar counselling with free NRT for 2 weeks. All subjects received printed self-help materials to support their quitting efforts. A structured questionnaire was used for data collection, including pattern of NRT use and self-reported 7-day point prevalence quit rate at 6 months and 12 months. Among the participants, the mean number of cigarettes smoked per day was 18.8 (SD = 10.9). By intention-to-treat analysis, 7-day point prevalence quit rates were not significantly different between A1 and A2 groups at 6-month (27.5% versus 27.3%; *P* = 0.97) and 12-month (21.1% versus 21.2%; *P* = 0.98) followup. The findings suggest that two-week free NRT was not more effective than 1-week free NRT to increase smoking cessation rate among Chinese smokers.

## 1. Introduction

Smoking and passive smoking are collectively the biggest preventable cause of death in Hong Kong, with major public health burden of morbidity, disability, and community costs [1]. Smoking cessation interventions with behavioral and pharmacological support are cost-effective [2–4]. However, there are constraints and variations in the provision of smoking cessation services in the primary health care setting [5, 6]. These included lack of trained smoking cessation counselors, resistance from policy makers to provide the service, and lack of funding to provide free pharmacotherapy. The United Kingdom (UK) National Health Service (NHS) started offering free supply of NRT since early 1998 under the NHS smoking cessation service [7]. The length of free NRT supply changed from 1 week in 1998 to full course through prescription since the publication of National Institute for Health and Clinical Excellence (NICE) guidelines in 2008 [8]. The NICE 2008 guidelines recommended to provide only for 2 weeks supply at the initial consultation, and subsequent prescriptions should be given only to people who have demonstrated, on reassessment, that their quit attempt is continuing [8]. The cost-effectiveness of this program was reported [9] and suggestions were made to continue the service on a regular basis [10]. The impact of the provision of free NRT ranging from one to twelve weeks was also reported in Canada [11], USA [12], and Australia [13], and it was argued that similar programs would support the promotion of smoking cessation service and quit rates.

The first Hong Kong Smoking Cessation Health Center (SCHC) provided free nicotine replacement therapy (NRT) for one week to the smokers who came for quitting services [14]. Clients were advised to buy NRT with their own money after the use of this initial free supply as there was no reimbursement mechanism for NRT through the medical insurance provider. Several previous studies reported that reimbursement for full course of 8–12 weeks NRT or other smoking cessation therapies would increase quit rates [3, 4, 15]. The United Kingdom [16] and United States [17] clinical practice guidelines recommend the use of NRT for a full course of 8–12 weeks or longer, but many healthcare providers cannot afford the full coverage, and many clients are unlikely to buy NRT after free supplies are finished [18]. In some operational settings, it is apparently not practicable to comply with the 8–12 weeks recommendation [19, 20]. Several studies reported that the offer of NRT at a reduced price or no cost increased both the use and cessation rates [21–23]. Evidence on the cost-effectiveness of increased NRT use with free supplies and cost sharing would be useful to support decisions about funding and coverage of smoking cessation services. Although the provision of a free full course of NRT would be an incentive to use quitting services, it might attract less motivated smokers with lower compliance in usage. This would result in increased expenditure and NRT wastage without improvement in quit rates. There is a need to identify effective, low cost interventions that would be acceptable and affordable in service settings with limited resources especially in low income countries, but there are no reports on randomized controlled trials to compare the effectiveness of providing different quantities of free NRT to smokers in a smoking cessation clinic.

In Hong Kong, several public and private health sector providers are offering new smoking cessation services now. The SCHC was the first regular but part-time service in Hong Kong, established in Ruttonjee Hospital in August 2000 by a multidisciplinary steering group based on the Hong Kong Council on Smoking and Health in collaboration with the Department of Community Medicine, The University of Hong Kong [14]. Based on the routine operation of the SCHC with one week free supply of NRT, we reported a higher quit rate among those who used NRT for 4 weeks or longer (40%) than those who used NRT for less than 4 weeks (25%) [20]. However, only 16% used NRT for 4 weeks or longer. Because withdrawal symptoms are higher during the first two weeks of quitting smoking [24, 25], ensuring the proper use of NRT during this initial period could help to increase quit rates. We hypothesized that if, for 2 weeks, supply of NRT could be given to smokers free of charge, more will continue using NRT and successfully quit. 

We aimed to increase the quit rate by providing one additional week free NRT. In our earlier analysis of the phase 1 data of our clinic, we found a higher 7-day point prevalence quit rate among those who used NRT for 2 weeks or longer (39%) than those who used NRT for one week only (23%) [20]. Therefore, the aim of this study was to examine if 2-week free NRT would be more effective than 1-week free NRT to help smokers quit smoking at 6 and 12 months. 

## 2. Methods

### 2.1. Study Population

The study took place in Hong Kong SCHC [14]. Subjects were current smokers who called the SCHC booking hotline to make an appointment and attended the SCHC during the study period. The smoking cessation counselor recruited eligible smokers for the trial. To be eligible for the trial, the subjects had to be (a) aged 16 or above, (b) able to speak Chinese, (c) smoking at least 5 cigarettes daily, (d) clearly motivated to quit, (e) willing to use NRT, (f) free from any serious health problems that may make them unsuitable for using NRT (e.g., recent stroke, palpitations, or other life threatening conditions), (g) not receiving other forms of smoking cessation programs, and (h) signatory to an informed consent form. Subjects were excluded if they did not meet the inclusion criteria or if they were on regular psychotropic medications. 

### 2.2. Study Design

This was a single-blinded randomized controlled trial (RCT) conducted in the Hong Kong SCHC. The study protocol was approved by the Ethics Committee of the Faculty of Medicine, the University of Hong Kong. 

### 2.3. Procedures

Eligible selected subjects signed the consent form and completed the baseline measures (filling in a questionnaire and measurements of body weight, height, blood pressure, and exhaled carbon monoxide) before the counselor opened a serially numbered, opaque, and sealed envelope (SNOSE) to reveal the random assignment of each smoker to A1 or A2 group. The random numbers for group assignment were generated by the research assistant (not the counselors) of the project using a personal computer before subject recruitment. Subjects in both groups received 20 min face-to-face individual smoking cessation counseling by the trained smoking cessation counselor and were provided with a 1-week or 2-week free supply of NRT (either gum or patches, according to subject's preference). Participants in the A1 group received 1-week free NRT, and the A2 group received 2-week free NRT supplies. The NRT was prescribed using the guideline based on the subject's daily cigarette consumption [17]. All participants received NRT after the initial counseling session. At initial contact, participants in both groups received a self-help quitting pamphlet, “Easy Steps to Quitting,” developed by the Hong Kong Council on Smoking and Health.

At 1 week, a telephone call was made to each participant to check whether they were facing any difficulties, and a brief counseling (less than 5 minutes) was given if requested. Subjects in both groups were contacted, face-to-face for all those who attended the SCHC and by telephone for all those who did not attend, at 1 and 3 months for a follow-up assessment and relapse prevention counseling. The standard questionnaire was used at each follow-up visit or other contacts. Each session lasted for an average of 15 minutes. To assess the final outcome, all subjects were again followed up at 6 and 12 months by telephone for an average of 10 minutes. An independent interviewer, who was unaware of the subject's group allocation, carried out the 6 and 12 months follow-up interview. At 6 months, those who had stopped smoking (not smoking for 7 days or more preceding the follow-up interview) were invited to attend the SCHC for biochemical validation by measuring carbon monoxide level in expired air. We offered an incentive of HK$200 (US$1 = HK$7.8) for attending the validation test. 

### 2.4. Intervention

The 20 min face-to-face smoking cessation counseling intervention included two components of the information on the health consequences of smoking and the smoking cessation counseling. Counselling was provided based on the queries and the needs of individual clients which were determined by their smoking status, physical dependence and the perceived barriers to quitting. A nondirective patient-centred intervention utilizing motivational interviewing techniques and the 5R (relevance, risk, rewards, roadblocks, and repetition) approach was used to boost motivation [26]. The smoking cessation counselor encouraged all smokers to set a quit date and provided tips to smokers for successful quitting and instructions to use NRT as appropriate. Details of the counseling method have been described in another report [14]. A hotline number was available to all study participants through which they could contact counselors to discuss any difficulties in their quitting process and to receive problem-oriented solutions. However, only a very small number called the hotline for advice. Participants in both groups were advised to buy NRT using their own money after finishing the free supply. The recommended duration of NRT use was 8–12 weeks depending on the NRT type (patch or gum) given [25]. 

All counselors were trained smoking cessation counselors, attended a 4-day training course on smoking cessation skills organized by the University of Hong Kong, and passed the final assessment examination. To ensure the quality of the intervention, regular meetings with the counselors were held every 2 months for case sharing and evaluation. 

### 2.5. Outcome Measures

The main outcome was self-reported 7-day point prevalence quit rate at 6 months (defined as not smoking during the 7 days preceding the 6-month followup). We used this as our primary outcome measure because this measure is most commonly used across smoking cessation studies. We have also reported the same measures in our earlier studies among the Hong Kong Chinese people [14, 20, 27]. 

To report the complete picture of the intervention, we have also measured several secondary outcome variables [14, 20, 27]: biochemically validated (expired carbon monoxide reading <9 ppm [28]) 7-day point prevalence quit rate at 6 months, 24-hour point prevalence quit rate at 6 and 12 months without validation (defined as not smoking during the 24-hours preceding the 6-month and 12-month followups), continuous quit rate at 6 and 12 months without validation (defined as continuously not smoking during the 6 months and 12 months preceding the 6-month and 12-month followups), and reduction rate (had not quit but had reduced smoking by at least 50% from the baseline level at 6 and 12 months) among those who were not able to quit and whether subjects had quitted for at least 24 hours at some point before 6-month and 12-month followup.

### 2.6. Sample Size and Power

Our sample size calculation was based on our earlier studies among the same clinic population [14, 20]. In the earlier report, we reported a higher quit rate among those who used NRT for 2 weeks (39%) versus those who used NRT for 1 week (23%) [20]. In another evaluation report, we have reported a quit rate of 27% (95% confidence interval, 25–30%) among the attendees of the SCHC [14]. Based on these previous results, for the current study, we intentionally adopted a conservative estimate and hypothesized that, by giving 2 weeks' free NRT supply, the quit rate would be 24% in the 1-week NRT group and would increase to at least 36% in the 2-week NRT group, with an absolute effect size of 12%. This yielded a total sample size of 248 in each group, based on a significance level of 5% and power of 90%. Assuming a 10% drop-out rate, we estimated a total sample size of 544 (272 in each group). We achieved a final sample size which was a bit higher than planned so as to obtain a greater power.

**Figure 1:**
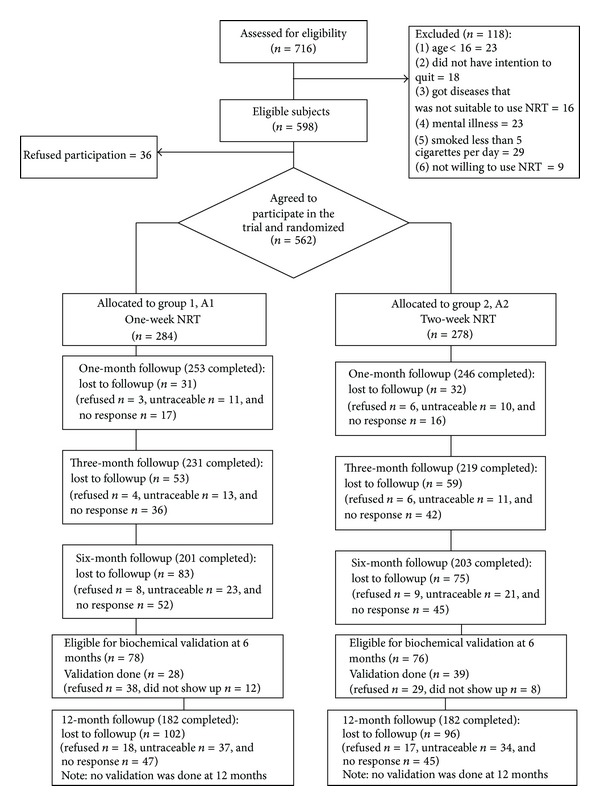
Flow of participants through trial.

All the 716 subjects who attended the clinic underwent screening for inclusion, and 118 were excluded. Of the 598 eligible subjects, 36 refused to participate, and 562 were available for randomization (Figure 1). 

### 2.7. Data Collection and Analysis of Data

A standardized structured questionnaire was used, the details of which have been described elsewhere [14, 20]. The questionnaire included demographic information (gender, marital status, age, occupational status, educational attainment, and household income) and tobacco use related information (smoking and quitting history, spouse or other household members' smoking status, nicotine dependency level, and stages of change). Carbon monoxide, using Bedfont piCO+ Smokerlyzer, in expired air was measured among those who attended the SCHC for biochemical validation at 6 months. At 6-month and 12-month follow-up contacts, participants were asked about the helpfulness of the smoking cessation counseling, the follow-up contacts, and the self-help materials. 

We analyzed the data with SPSS for Windows, version 10.0. The baseline characteristics of two groups (A1 and A2) were compared by Chi-square test. To test the efficacy of the two different lengths of NRT use, the two groups were compared. Rates of tobacco abstinence and reduction between groups were compared using Pearson's Chi-square test, together with odds ratios (OR) and their 95% confidence intervals (CI). We conducted all analyses on an intention-to-treat basis. Follow-up variables with missing data were set to their baseline values (e.g., current smoker). A two-tailed *P* value of less than 0.05 was considered statistically significant.

## 3. Results

### 3.1. Baseline Characteristics

Of the 562 subjects, most were males (73.3%), married or cohabiting (52.8%), had attained education to secondary school level or above (90.4%), and were currently employed (75.6%). The mean number of cigarettes smoked per day was 18.8 (SD = 10.9), and the average number of years of smoking was 18.5 (SD = 10.9). No significant differences were found between groups.  Table 1 shows that the A1 and A2 groups were similar in their demographic and other variables. 

### 3.2. Pattern of NRT Use

Overall, less than half of the subjects used NRT for at least 2 weeks, and only about a quarter used it for at least 4 weeks. All of those who could not be contacted were considered as non-users (Table 2). There was no significant difference in the patterns of NRT use between subjects in the A1 and A2 groups except a higher proportion of those in the A2 group who used NRT for at least 2 weeks (53.6% versus 41.5%) (*χ* ^2^ = 0.54, df = 1, *P* = 0.004).

### 3.3. Quitting Outcome

Table 3 shows that, at 6-month followup, the self-reported 7-day point prevalence quit rate was similar in the A1 group (27.5%; 78/284) and the A2 group (27.3%; 76/278) (OR = 1.0, 95% CI: 0.7–1.4, *P* = 0.97). The self-reported quit rate at 12 months was also almost similar in groups A1 (21.1%; 60/284) and A2 (21.2%; 59/278) (OR = 1.0, 95% CI: 0.7–1.5; *P* = 0.98). The biochemically validated quit rate at 6 months was higher in group A2 (12.6%; 35/278) than in group A1 (7.7%; 22/284), but the difference was not statistically significant (OR = 1.7, 95% CI: 0.9–3.0). We also found no significant differences in other secondary outcome measures between the two groups at 6 months and 12 months (Table 3). 

### 3.4. Other Exploratory Outcomes

In relation to the satisfaction with the intervention, participants overwhelmingly found the cessation counseling helpful (94.8%), the follow-up contacts useful (91.2%), and the self-help quitting materials useful (89.3%). We have explored the quit rate based on the type of NRT use and found that patch users and gum users did not differ in the quitting outcome at both 6 months and 12 months (*P* > 0.05) (data not shown). In relation to the call to the counselor's hotline, only eight participants (group A1 = 5 and group A2 = 3) called the hotline, and all the calls were for more NRT supply and general enquiry. Due to the small number, we did not explore the quitting outcome based on the calling status to the hotline.

## 4. Discussion

Our findings showed that giving 2 weeks' free NRT over 1 week's free NRT did not increase quit rate at 6 months and 12 months among Chinese smokers attending a smoking cessation clinic in Hong Kong. Although a higher proportion in the A2 group used NRT for at least 2 weeks probably due to the free supply, there was no impact on the reported overall quit rates. One possible explanation was that giving 1-week or 2-week free NRT did not change smokers' adherence to NRT use (defined as NRT use for 4 weeks or longer [20]), as the prevalence of NRT use was almost identical in both groups, (22.9% in A1 group versus 24.8% in A2 group). Another explanation was that 1-week free NRT was enough to encourage motivated smokers to continue to use it for a longer duration, because some smokers who attended the clinic were already motivated to quit and giving 1-week or 2-week supply did not have any measurable effect on their determination to quit and buy NRT afterwards. This further reflects the importance of self-motivation in quitting smoking [29]. Also, the price of NRT as compared to the price of the cigarettes is an important consideration for smokers. In Hong Kong, during the study period, the price of one-week NRT (US$38) was similar to the price of 8 packs of cigarettes. Therefore, buying NRT rather than cigarettes did not provide any immediate financial incentives to the participants. 

Whether the suggestion that full free coverage for smoking cessation service including both behavioral counseling and NRT [3, 4] would be more beneficial for Chinese smokers than partial coverage for NRT needs to be further tested. Although the United States Clinical Practice Guideline *Treating Tobacco Use and Dependence* recommends that health service organizations cover the cost for NRT [25], our results suggest that increased coverage to two weeks may not be more effective than a minimal period of one week. Because of the high cost of NRT, initial support for free NRT only for 1 week would be appropriate for services which are not well funded. Motivated smokers should be encouraged to buy and use NRT after trying the free NRT for 1 week. Providing nonmotivated or nonadherent smokers more free NRT would result in wastage and would not increase NRT usage rate or quit rate. One study found that the percentage of the amount used was the inverse of the amount provided [30]. That is, if a caller was given a 1- or 2-week supply of medication, they would more likely use a larger percentage of it than those given a 6-week supply [30]. Several previous reports and data on wastage are often not reported by service providers offering full NRT coverage [12, 31], and further revaluation on wastage and cost-effectiveness is needed.

The study had several limitations. Because followup lasted for only 12 months, results on longer term quit rate were not available. Quitting was not verified biochemically at 12 months, because we measured main outcomes at 6 months and two-third did not return for validation. However, validation is not required in low-intensity population-based interventions such as the present study [32]. Another limitation was that some participants were lost to followup despite our active efforts which might have weakened the effectiveness of the intervention. Also, our sample size calculation was based on the anticipated dropouts at 6 months only. This is because we did not plan to conduct 12-month followup at the initial stage, but finally we were able to conduct a followup at 12 months. We thought reporting both 6 and 12 months results will provide a complete picture of the study. Finally, intent-to-treat analysis tends to be conservative and underestimate the effect size. 

Our study should have important practical implications. As the first study showing effectiveness of one-week free NRT, our findings can help policy makers to budget for NRT costs in smoking cessation services and for insurance companies to consider reimbursement to cover NRT expenditure for insured smokers. Our study also provides evidence from an East Asian population that a behavioral counseling program together with minimal free provision of NRT can produce a satisfactory quit rate, which is comparable to or higher than other programs providing full coverage for NRT [2, 6]. 

The optimal duration to provide tobacco cessation medication is still undefined. The USPHS Guideline varies from no more than 8 weeks for nicotine patch to up to 12 weeks for nicotine gum, nicotine lozenge, bupropion SR, and varenicline [33]. The Centers for Disease Control and Prevention (CDC) recommends quitlines that provide a minimum of two weeks for all callers and up to 8 weeks for those financially disadvantaged [34]. Bauer et al. [35] argued that the individual's experience with tobacco cessation medication may increase the awareness of the benefits of these medications and the utilization of smoking cessation service for future quit attempts—especially for those who may have never used either intervention before. In another study, as little as one week of free NRT increased calls to a quitline and increased tobacco abstinence rates compared to not providing NRT [30]. However, many smokers can quit successfully only with behavioral counseling [36]; over-emphasis on NRT or other medications should be avoided. Although linking free tobacco cessation medications for all cessation support seekers with counseling ensures that more tobacco users will receive both types of interventions [37] to achieve a greater likelihood of abstinence [17]; in many developing countries, this may not be feasible [5]. Service providers in many developing countries often cannot afford any free NRT or other smoking cessation medications and would rely on advice or counseling only. Therefore, specific smoking cessation program should be customized based on the resources available and the feasibility of the intervention within the given healthcare setting. However, there would be a need for rigorous evaluation of any pilot programs before their wider implementation to the population level.

In conclusion, this study demonstrates no additional advantage of offering free NRT for 2 weeks as compared with 1 week to Chinese smokers in relation to self-reported or biochemically validated quitting smoking. Provision for counseling and/or free NRT or other cessation medications should be based on the availability of resources of a specific program. However, limited course of medication for brief periods of time (i.e., for 1 week) as a promotional tool encourages smokers to seek cessation support and may increase quit rate [30].

## Figures and Tables

**Table 1:** Baseline demographic, smoking, and other characteristics of the two groups of participants.

Characteristics	A1 (*n* = 284) % (*n*)	A2 (*n* = 278) % (*n*)	*P* value for *χ* ^2^ test
*Demographics*:			
Gender			0.84
Male	77.8 (221)	78.8 (219)	
Female	22.2 (63)	21.2 (59)	
Marital status			0.57
Single	39.4 (112)	42.1 (117)	
Married/cohabiting	53.2 (151)	52.5 (146)	
Divorced/separated/widowed	7.4 (21)	5.4 (15)	
Occupational status			0.20
Currently employed	74.3 (211)	77.0 (214)	
Unemployed	9.9 (28)	12.9 (36)	
Housewife	8.1 (23)	4.0 (11)	
Full-time student	3.5 (10)	3.2 (9)	
Retired	4.2 (12)	2.9 (8)	
Age, years			0.77
25 or below	16.2 (46)	17.3 (48)	
26–35	39.8 (113)	35.6 (99)	
36–45	24.6 (70)	25.5 (71)	
46 or above	19.4 (55)	21.6 (60)	
Educational attainment			0.56
Primary as below	9.9 (28)	9.4 (26)	
Secondary (F1–F5)	61.3 (174)	57.6 (160)	
Matriculation or above	28.9 (82)	33.1 (92)	
Monthly household income			0.93
HK$9,999 or less	22.5 (64)	21.6 (60)	
HK$10,000–29,999	56.3 (160)	56.1 (156)	
HK$30,000 or above	21.1 (60)	22.3 (62)	

*Tobacco use related: *			
Daily cigarette consumption in the past month			0.30
10 or less	27.8 (79)	25.5 (71)	
11–20	46.5 (132)	52.9 (147)	
21 or above	25.7 (73)	21.6 (60)	
Nicotine dependency level^†^			0.58
Low	26.1 (74)	28.4 (79)	
Moderate	32.7 (93)	28.8 (80)	
Severe	41.2 (117)	42.8 (119)	
Years of smoking			0.83
1–10	28.2 (80)	26.3 (73)	
11–20	36.6 (104)	36.3 (101)	
20 or more	35.2 (100)	37.4 (104)	
Spouse or household members' smoking status			0.82
Smoker	12.3 (35)	12.9 (36)	
Nonsmoker	87.7 (249)	87.1 (242)	
No. of previous quitting attempt (s)			0.55
None	28.5 (81)	26.3 (73)	
One or more	71.5 (203)	73.7 (205)	
Length of abstinence in the last quitting attempt*			0.08
1–30 days	71.4 (145)	79.0 (162)	
>30 days	28.6 (58)	21.0 (43)	
Stage of change			0.18
Precontemplation	6.3 (18)	5.8 (16)	
Contemplation	71.8 (204)	64.7 (180)	
Preparation	18.0 (51)	25.5 (71)	
Action	3.9 (11)	4.0 (11)	
Choice of NRT use			0.65
Patch	71.2 (202)	69.0 (192)	
Gum	28.8 (82)	31.0 (86)	

Note: US$1 = HK$7.8.

^†^Nicotine dependence level was measured by Fagerstrom scale. It is divided into 3 levels: low (score 0–3), moderate (score 4-5), and severe (score = 6–10).

*This question is only for those subjects who had attempted quitting smoking in the past.

**Table 2:** NRT use among subjects in the two groups.

Duration of NRT use	A1 (*n* = 284)	A2 (*n* = 278)
2 weeks or more	41.5*	53.6*
4 weeks or more	22.9	24.8
8 weeks or more	7.4	6.8

Note: all of those who were not available at followup were considered as nonusers.

*Significant difference between the two study groups (Chi-square = 0.54, df = 1, *P* = 0.004).

**Table 3:** Quitting status using different outcome measures in the two groups at 6-month and 12-month followups, by intention to treat*.

Outcome measures	6 months				12 months			
	A1 (*N* = 284) *N* (%)	A2 (*N* = 278) *N* (%)	*P* values	OR (95% CI)	A1 (*N* = 284) *N* (%)	A2 (*N* = 278) *N* (%)	*P* values	OR (95% CI)
*Main outcome *								
Self-reported 7-day point prevalence quit rate	78 (27.5)	76 (27.3)	0.97	1.0 (0.7–1.4)	60 (21.1)	59 (21.2)	0.98	1.0 (0.7–1.5)

*Secondary outcomes *								
Biochemically validated (CO level in exhaled air) 7-day point prevalence quit rate	22 (7.7)	35 (12.6)	0.06	1.7 (0.9–3.0)	NA	NA	NA	NA
Self-reported 24-hour point prevalence quit rate	78 (27.5)	76 (27.3)	0.97	1.0 (0.7–1.4)	60 (21.2)	59 (21.2)	0.98	1.0 (0.7–1.5)
Self-reported continuous abstinence	71 (25.0)	69 (24.8)	0.96	1.0 (0.7–1.5)	52 (18.3)	51 (18.3)	0.99	1.0 (0.7–1.5)
Had not quit but had reduced smoking by at least 50% from the baseline level	49 (17.3)	50 (18.0)	0.48	1.1 (0.7–1.9)	39 (13.7)	44 (15.8)	0.48	1.2 (0.7–1.9)
Stopped smoking for at least 24 hours at some point prior to the interview	123 (47.9)	127 (53.1)	0.24	1.2 (0.9–1.8)	110 (38.7)	112 (40.3)	0.71	1.1 (0.8–1.5)

Note: OR: odds ratio; CI: confidence interval; NA: not applicable.

*Subjects who did not complete the intervention (withdrawn/could not be contacted) were considered not quitting. Those who had no validation were also considered as not quitting.
